# Undifferentiated High-Grade Pleomorphic Sarcoma of the Posterior Mandible: A Report of a Rare Case

**DOI:** 10.7759/cureus.101947

**Published:** 2026-01-20

**Authors:** Nivetha D., Christeffi Mabel, Abimathi R., Varshaa Ramesh

**Affiliations:** 1 Oral Medicine and Radiology, Chettinad Dental College and Research Institute, Chennai, IND

**Keywords:** connective tissue neoplasm, giant-cell–rich lesion, mandible, sarcoma, undifferentiated pleomorphic sarcoma, ups

## Abstract

Undifferentiated pleomorphic sarcoma (UPS) is a high-grade mesenchymal malignancy that can closely resemble benign giant-cell-rich lesions such as central giant cell granuloma (CGCG), especially on limited biopsies. We report a case of a female patient with an enlarging mandibular swelling and an expansile osteolytic lesion that was initially diagnosed as CGCG based on an incisional biopsy showing multinucleated giant cells in a fibroblastic stroma. However, extended immunohistochemistry and the aggressive radiologic features supported a revised diagnosis of giant-cell-rich variant UPS. This case highlights the potential for diagnostic confusion on insufficient samples and emphasizes the importance of adequate tissue sampling, correlation with imaging, and comprehensive ancillary testing when evaluating destructive mandibular lesions.

## Introduction

About 1% of all head and neck cancers are sarcomas of the craniofacial skeleton [1]. There are different types of sarcomas occurring in the oral cavity involving both soft tissues and bone. Sarcomas involving bone include osteosarcoma and Ewing sarcoma, while those affecting the soft tissues include leiomyosarcoma, rhabdomyosarcoma, and undifferentiated sarcoma [2]. In most patients with soft tissue sarcomas (STS), no definitive etiologic factor can be identified. However, prior exposure to therapeutic ionizing radiation or certain chemical carcinogens or any alkylating chemotherapeutic agents has been associated with sarcomagenesis [3]. More recently, specific genetic and molecular alterations have also been associated with the pathogenesis of STS [3]. Undifferentiated/Unclassified sarcomas include tumors in which all identifiable lines of differentiation have been excluded. Many of these neoplasms, particularly those with pleomorphic features, were previously classified as malignant fibrous histiocytoma. They are generally high-grade lesions, exhibit marked morphological heterogeneity, and are frequently associated with an unfavorable prognosis. Based on the predominant morphological pattern, these tumors may be subclassified into round cell, spindle cell, pleomorphic, and epithelioid types [4]. Undifferentiated pleomorphic sarcoma (UPS) is incredibly uncommon in the oral cavity. About 5% of adult STS are undifferentiated high-grade pleomorphic sarcomas (UPS), which are more frequently found in the extremities, mainly the lower limbs, and are also reported in some cases involving the retroperitoneum. It is more common in elderly patients during the sixth and seventh decades of life [5]. From a clinical standpoint, they usually manifest as rapidly progressive masses, often with surface ulceration and no characteristic gross appearance [6]. Imaging modalities such as computed tomography (CT) and cone-beam computed tomography (CBCT) provide three-dimensional evaluation of the true extent of the lesion, accurately demonstrating cortical expansion, thinning or perforation, internal architecture, and involvement of adjacent anatomical structures, which may not be fully appreciated on conventional radiographs [7]. However, because aggressive lesions can show overlapping imaging and routine histologic features, extended immunohistochemistry and, where indicated, next-generation sequencing (NGS) are often required to resolve diagnostic uncertainty and establish a definitive diagnosis [8]. Diagnosis of UPS poses significant challenges, as it lacks unique clinical and histopathological hallmarks. The lesion often presents as a rapidly enlarging mass that may be painless or associated with nonspecific discomfort, and can mimic a range of benign, reactive, or other malignant soft tissue conditions, leading to potential delays in diagnosis. In the present case, a similar diagnostic dilemma was encountered, wherein the initial histopathological examination suggested a diagnosis of central giant cell granuloma; However, subsequent immunohistochemical analysis and excision of the lesion in toto confirmed a high-grade sarcoma with undifferentiated features.

The purpose of this case report is to document a rare manifestation of high-grade UPS involving the posterior mandible, detailing its clinical, imaging, and microscopic characteristics, while emphasizing the key diagnostic considerations and the importance of early recognition in guiding appropriate management.

## Case presentation

Patient history

A 56-year-old female patient presented with a complaint of swelling in the right lower posterior jaw region for approximately four months, which developed following an uneventful extraction of a mandibular posterior tooth. The swelling was insidious in onset and gradually progressive, associated with intermittent, mild pain. There was no history of paresthesia, fever, weight loss, or constitutional symptoms. The patient also denied any history of prior trauma, radiation exposure, or systemic illness, and had no significant medical comorbidities or any deleterious oral habits.

Local and regional examination

**Figure 1:**
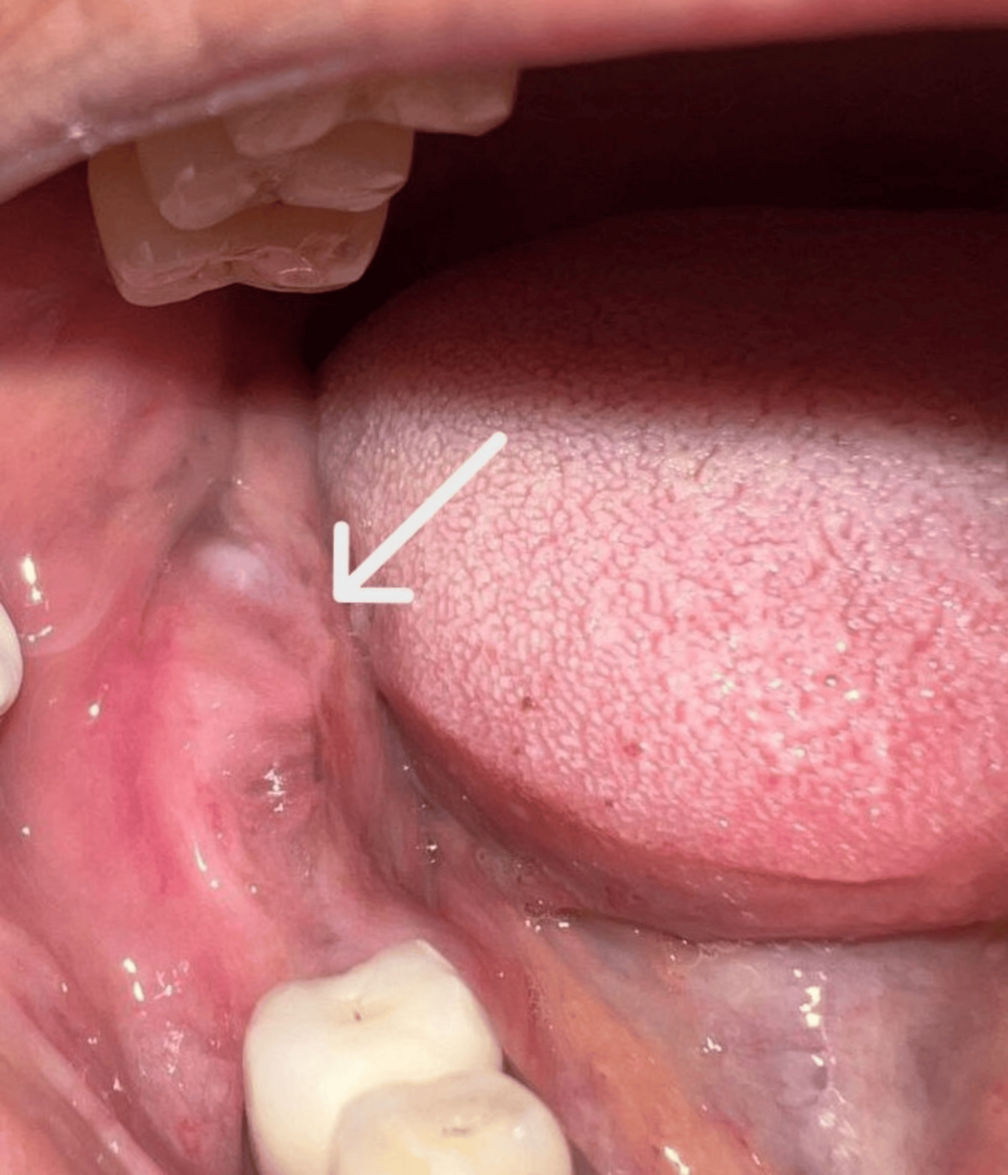
Intraoral view. The preoperative intraoral photograph reveals a diffuse swelling along the right posterior edentulous ridge. The arrow indicates the area of mucosal elevation with subtle erythema and surface smoothness, corresponding clinically to the underlying lesion.

Intraoral examination showed a diffuse swelling measuring approximately 3 × 1 cm in the right posterior edentulous mandibular region, extending mediolaterally along the alveolar mucosa and anteroposteriorly from distal of 45 to the retromolar area (Figure 1). The surface was smooth, and the color was similar to that of the adjacent mucosa. On palpation, the lesion was very soft in consistency, tender, and compressible. Regional lymph node examination, including submandibular and cervical nodes, revealed no clinically palpable lymph nodes. Although the presenting symptoms were nonspecific, the rapid progression of the lesion following tooth extraction in the posterior mandible raised clinical concern. As the posterior mandible is a recognized predilection site for metastatic lesions as well as odontogenic cysts and tumors, cystic pathologies were also included in the differential diagnosis.

Radiographic evaluation

**Figure 2:**
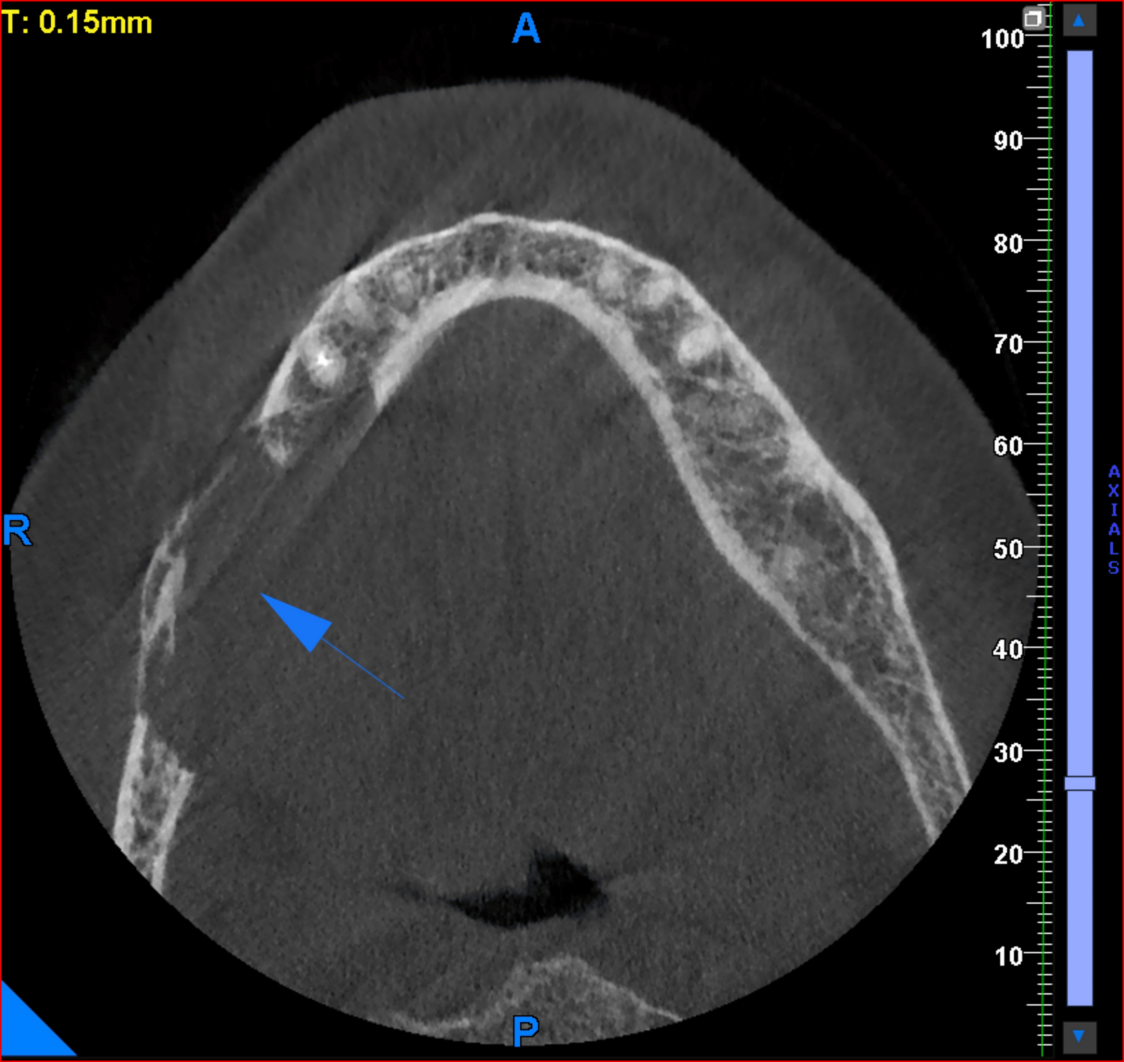
CBCT: axial section The arrow indicates an expansile osteolytic lesion with irregular margins involving the alveolar process of the right mandible, with erosion of both buccal and lingual cortical plates. CBCT, cone-beam computed tomography

**Figure 3:**
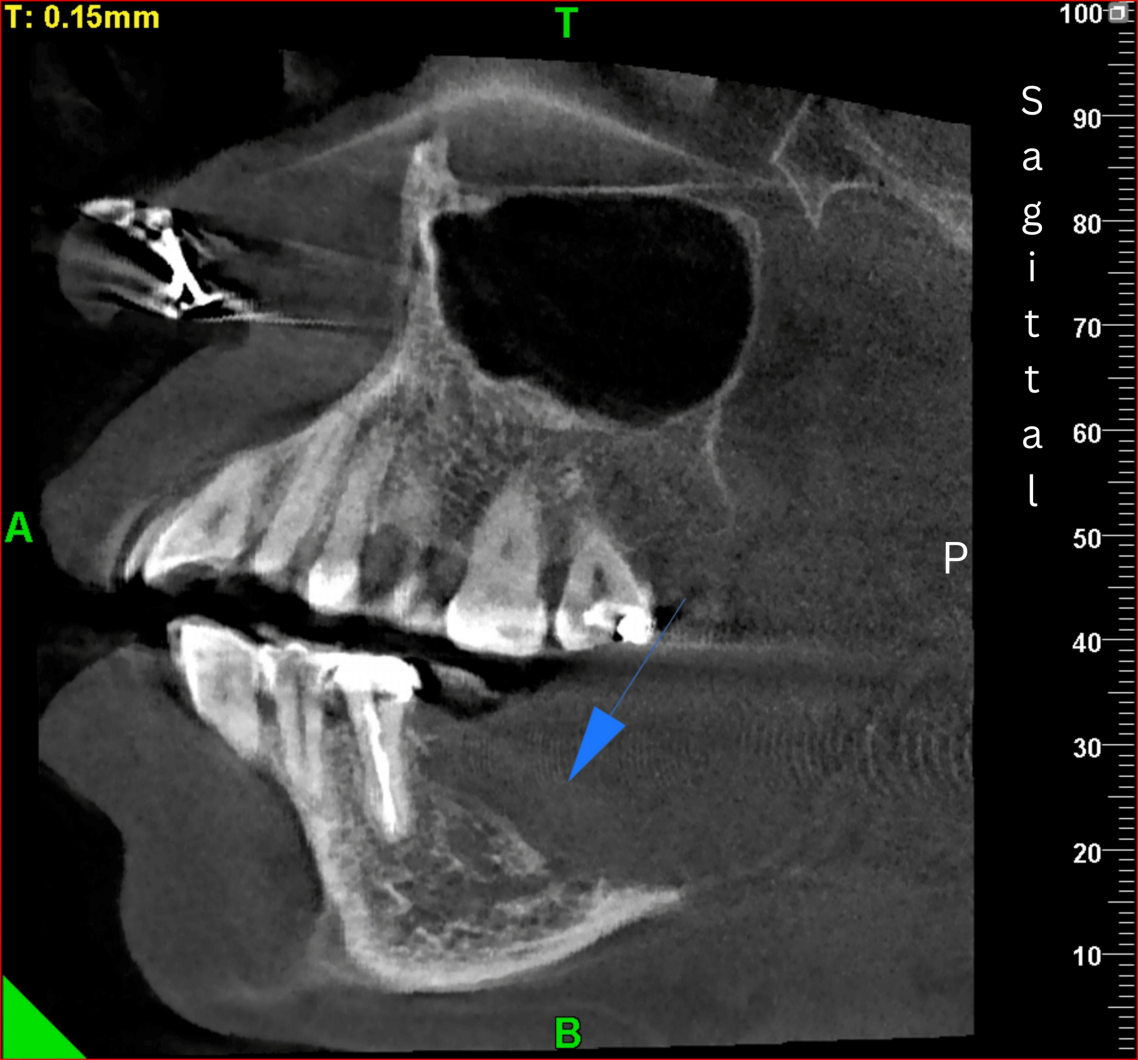
CBCT: sagittal section. The arrow demonstrates the involvement of the body and ramus of the right posterior mandible with destruction of cortical bone and alteration of the surrounding trabecular pattern. CBCT, cone-beam computed tomography

As a part of the radiographic evaluation, cone-beam computed tomography (CBCT) was performed. CBCT revealed an expansile osteolytic lesion with irregular margins involving the alveolar process, body, and ramus of the right posterior mandible, with erosion of both the buccal and lingual cortical plates (Figures 2-3). 

Incisional biopsy and initial histopathologic findings

**Figure 4:**
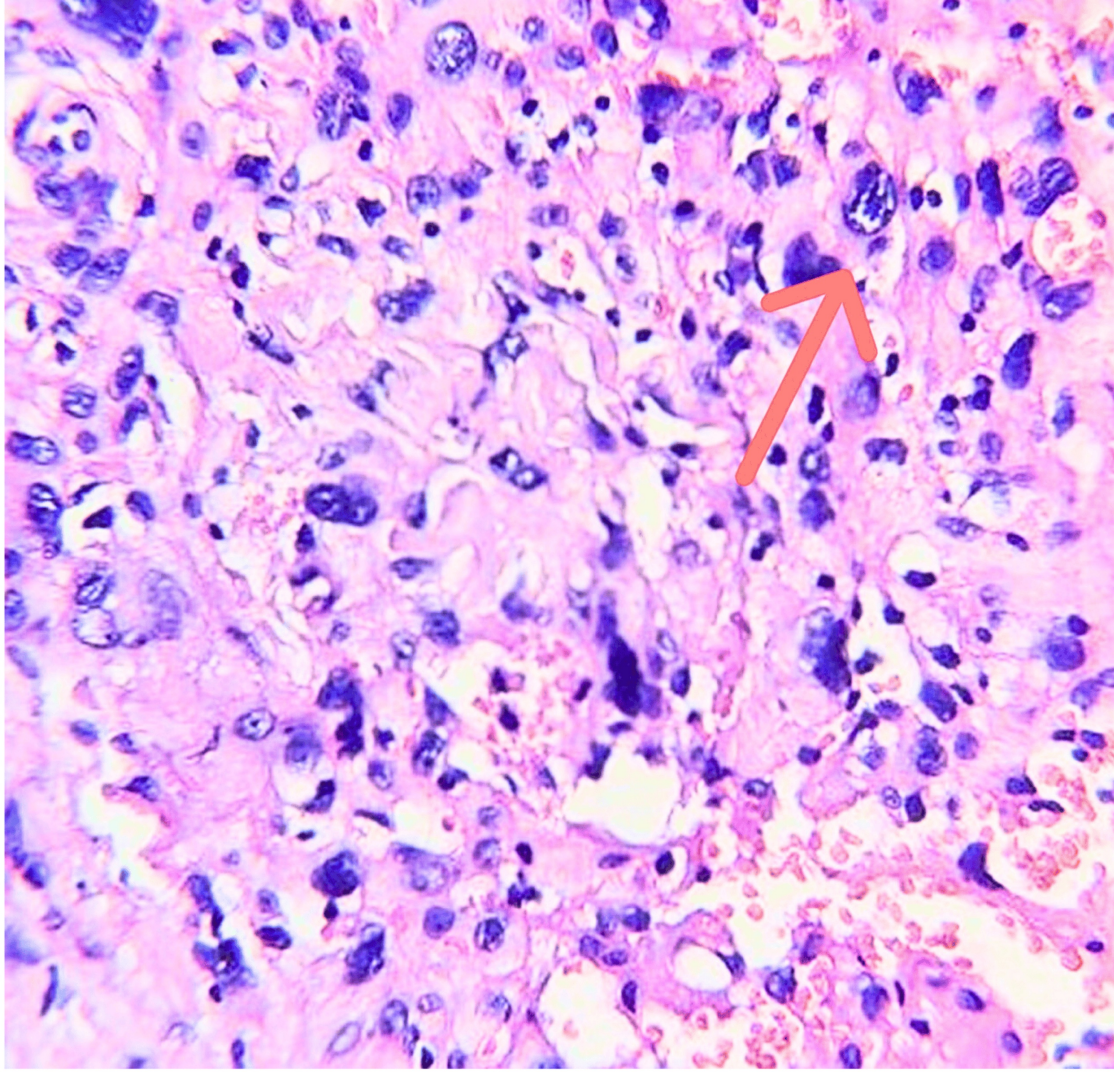
Photomicrograph of the incisional biopsy specimen showing unevenly distributed multinucleated giant cells (arrow) (H&E stain, original magnification ×100). H&E, hematoxylin and eosin

Within two weeks of initial presentation, an incisional biopsy was subsequently performed from the posterior mandibular region distal to the last tooth. Multiple tissue fragments were obtained (five fragments measuring approximately 0.4-0.6 cm each) and submitted for histopathological examination. Microscopic evaluation revealed fragments of tissue composed of spindle-shaped cells arranged in fascicles, admixed with unevenly distributed multinucleated giant cells and a lymphocytic inflammatory infiltrate. Some cells exhibited hyperchromatic, irregular nuclei with moderate to abundant cytoplasm (Figure 4). Bony trabeculae embedded within fibrous stroma, focal adipose tissue fragments, and thin- and thick-walled vascular spaces were also noted. No mitosis/necrosis were seen. Due to the presence of multinucleated giant cells and the absence of overt malignant features in the incisional biopsy, the findings were considered with a broad differential diagnosis of giant cell-rich lesions. As the incisional biopsy was inconclusive, further immunohistochemical evaluation was advised.

Adjunctive investigations

**Figure 5:**
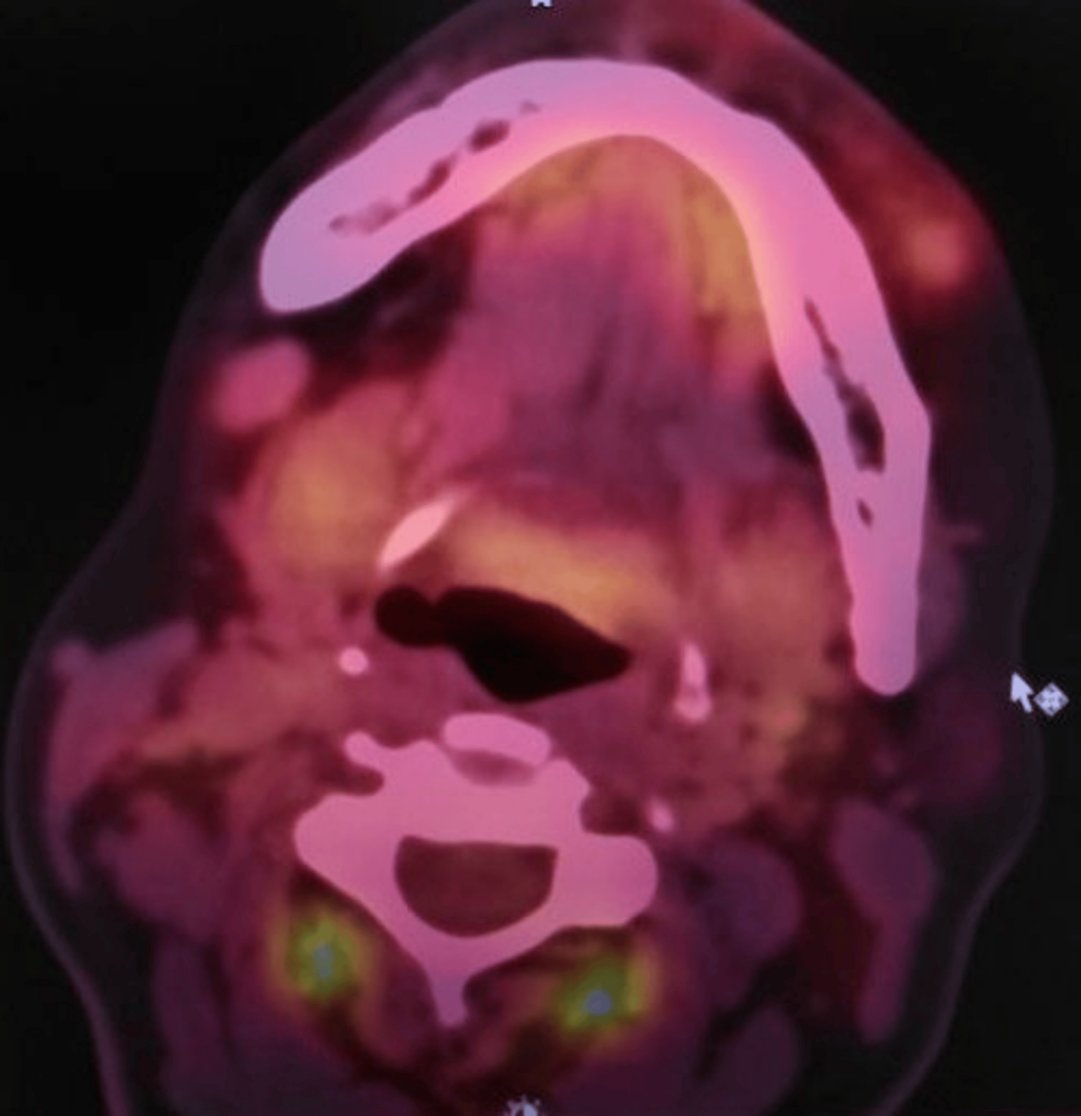
PET-CT image. A focal FDG-avid lesion is noted in the right posterior mandible, showing increased metabolic activity corresponding to the primary site of involvement. The bilateral submandibular lymph nodes are subcentimetric, non-FDG-avid, and non-hypermetabolic. PET-CT, positron emission tomography-computed tomography; FDG, fluorodeoxyglucose

Meanwhile, positron emission tomography-computed tomography (PET-CT) was performed to assess the metabolic activity of the lesion and to exclude a primary tumor or distant metastasis. The lesion demonstrated fluorodeoxyglucose (FDG) avidity limited to the right posterior mandible, with no evidence of metabolically active disease elsewhere (Figure 5). Subsequently, the immunohistochemical results revealed the following: Pan CK negative, CD68 positive in scattered cells, desmin negative, Ki-67 20%. Clones used were Pan-cytokeratin (AE1/AE3), desmin (DER11), and Ki-67 (MM1). However, the results were only suggestive and not definitive due to limited biopsy material, providing a diagnosis of central giant cell granuloma. In view of the aggressive radiologic features and the discordance between imaging findings and the initial histopathologic impression, further diagnostic evaluation was considered necessary.

Definitive surgical management

Following further evaluation, definitive surgical management was undertaken. One week after immunohistochemical assessment and multidisciplinary discussion, the patient underwent right composite resection with right hemimandibulectomy, along with resection of the ipsilateral submandibular gland, sublingual gland, medial pterygoid muscle, and masseter muscle.

Extended immunohistochemical analysis

Histopathological examination of the excised specimen revealed a high-grade sarcoma with marked cellular pleomorphism. The specimen was referred for further evaluation using a sarcoma immunohistochemical panel. Extended immunohistochemistry showed a purely mesenchymal phenotype with diffuse vimentin positivity and CD68 reactivity in multinucleated giant cells. No epithelial (pan-cytokeratin, CK-HMW, EMA, p40, p63), melanocytic (S100, HMB45), myogenic (SMA, desmin, myogenin), vascular (CD34), or neural markers were expressed. SATB2 showed only patchy, non-contributory staining. The Ki-67 index was approximately 18%-20%. The Ki-67 labeling index was assessed by estimating the percentage of positively stained tumor nuclei in areas of highest proliferative activity (Table 1). This favored a diagnosis of UPS, giant-cell-rich variant, after exclusion of specific lineages.

**Table 1:** Immunohistochemistry report. S-100 protein, calcium-binding protein family of neural crest origin; CD34, cluster of differentiation 34; HMB45, human melanoma black 45; Myogenin, myogenic differentiation protein; SMA, smooth muscle actin; Desmin, intermediate filament protein; Factor XIIIa, coagulation factor XIIIa; EMA, epithelial membrane antigen; PR, progesterone receptor; Ki-67, Kiel 67 (cell proliferation marker); ALK (D5F3), anaplastic lymphoma kinase (D5F3 antibody clone); Pan CK, pancytokeratin; Vimentin, mesenchymal intermediate filament protein; CD68, cluster of differentiation 68 (macrophage marker); p40, specific antibody detecting the p40 protein; p63, tumor protein p63; CK (high molecular weight), cytokeratin high molecular weight; SATB2, sequence binding protein 2

Markers	Result
S100	Negative
CD34	Negative
HMB45	Negative
Myogenin	Negative
SMA	Negative
Desmin	Negative
Factor XIIIa	Patchy positive
EMA	Negative
PR	Negative
Ki-67	18%-20%
ALK (D5 F3)	Negative
Pan-cytokeratin	Negative
Vimentin	Diffusely positive
CD68	Positive in giant cells
p40	Negative
p63	Negative
CK (high molecular weight)	Negative
SATB2	Patchy positive

Postoperative management and follow-up

**Figure 6:**
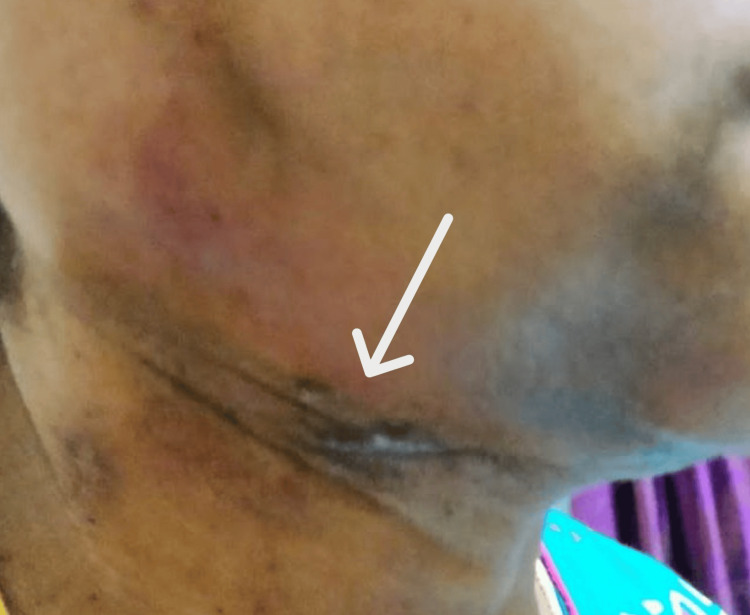
Postoperative image. One-month follow-up extraoral photograph showing a well-healed surgical incision (arrow) in the right submandibular region.

Postoperatively, the patient was planned for adjuvant radiotherapy, with chemotherapy proposed based on final staging and oncologic assessment. At the time of this report, the patient is under active oncologic follow-up with no evidence of local recurrence or distant metastasis during the short-term follow-up period (Figure 6).

## Discussion

Sarcomas of the oral cavity are rare, comprising approximately 1% of all malignant oral tumors [1]. Within the head and neck region, mandibular involvement accounts for a small proportion of sarcomas, estimated at 4%-10% [2]. STS represent a heterogeneous spectrum of malignant neoplasms derived from mesenchymal soft tissues [3]. Undifferentiated/unclassified sarcoma is a relatively recent category, which was formerly known as Malignant Fibrous Histiocytomas. It may account for up to 20% of pleomorphic soft tissue sarcomas, and approximately 25% of radiation-associated sarcomas [4]. According to the World Health Organization (WHO), a classification system of bone and soft tissue tumors with detailed cytogenetic and molecular profiles was proposed. The undifferentiated/unclassified sarcomas are grouped into the following categories: (1) undifferentiated spindle cell sarcoma, (2) UPS (previously classified as malignant fibrous histiocytoma), (3) undifferentiated round cell sarcoma, (4) undifferentiated epithelioid sarcoma, and (5) undifferentiated sarcoma not otherwise specified [4].

The current WHO classification includes this undifferentiated, unclassifiable category of pleomorphic sarcomas, encompassing high-grade malignant tumors in which no specific line of differentiation can be identified [5]. UPS accounts for approximately 5% of adult soft tissue sarcomas and most commonly involves the extremities [6]. In the study by Mura et al., it was suggested that this condition demonstrates a male predilection (M: F = 1.8:1), with reported patient ages spanning 8 to 88 years. A notable geographic clustering has been observed in Middle-Eastern and Asian regions, with the highest incidence documented in India (33.3%), followed by China (18.5%), Japan (11.1%), and Iran (5.6%) [6].

The etiology of UPS remains poorly understood. Proposed contributing factors include genetic susceptibility, environmental influences such as prior trauma or radiotherapy, and malignant transformation of preexisting benign lesions [9]. UPS may arise from both soft tissues and osseous structures; within the oral cavity, the mandible constitutes the predominant site of involvement (40.7%), followed by the maxilla (25.9%) [9]. UPS may arise in either soft tissue or bone at virtually any anatomical site, with a predilection for the extremities and retroperitoneum [9], with peak incidence in the sixth and seventh decades [10]. UPS has been reported in various head and neck sites, including the buccal mucosa, tongue, gingiva, maxilla, mandible, temporomandibular fossa, paranasal sinuses, salivary glands, and retro-orbital soft tissue. It is an aggressive neoplasm with a high propensity for distant metastasis [10].

UPS often tends to have an insidious onset with a rapidly enlarging mass, which is frequently painless but may be associated with nonspecific symptoms such as pain or paresthesia at the affected site as the lesion advances [10]. Symptoms may remain absent for prolonged periods, with lesions often detected incidentally or only after reaching a size that produces discomfort or functional limitation. Pain or neurological manifestations can occur secondary to mass effect or compression of adjacent neurovascular structures, while constitutional symptoms are uncommon and typically associated with metastatic disease [10]. UPS was historically difficult to evaluate radiologically, but significant progress has been made with the use of cross-sectional imaging. CT and MRI now offer valuable diagnostic insights [11]. On T1-weighted sequences of MRI, these lesions typically demonstrate low to intermediate signal intensity, often comparable to adjacent skeletal muscle. On T2-weighted images, they generally appear hyperintense, frequently with marked internal heterogeneity. Areas of intratumoral hemorrhage may be identified as regions of high signal intensity on T1-weighted images. Calcifications, reported in approximately 5%-20% of cases, are best detected on computed tomography but may occasionally be visualized on MRI as foci of low signal intensity across all sequences [11]. The rarity and biological heterogeneity of these tumors further complicate diagnosis, increasing the risk of misinterpretation and potentially compromising treatment planning and patient outcomes [12]. Earlier research underscored the need for robust diagnostic markers, which molecular pathology has since addressed by defining genetic signatures that correlate with distinct soft tissue sarcoma subtypes. NGS offers a comprehensive molecular characterization of tumors, enabling the identification of actionable genetic alterations that may be amenable to targeted or investigational therapies [12]. Immunohistochemical profiling plays a critical role in establishing the diagnosis of odontogenic sarcoma. Cytokeratin positivity helps identify residual epithelial nests and thereby excludes purely mesenchymal sarcomas. The sarcomatous component typically demonstrates immunoreactivity for CD34, vimentin, p53, and Ki-67, while lacking expression of S-100, smooth muscle actin, desmin, CD68, and CD117 [13]. As compared to other giant cell-rich lesions, incorporation of NGS, immunohistochemistry for mutation-derived proteins, and fluorescence in situ hybridization (FISH) into the diagnostic workup for head and neck giant cell-containing lesions may help prevent misclassification of rare but distinct entities as central giant cell granulomas [14].

The management of such sarcomas emphasizes wide-local excision with negative margins as the cornerstone of therapy. Incomplete resections, larger tumor size, deep location, and involvement of important structures (nerves, vessels) are established adverse prognostic factors [15]. Radiation therapy (pre- or postoperative) is often used, especially for large or deep tumors. Chemotherapy (e.g., doxorubicin-based) may be considered in high-risk or metastatic disease [16].

The case presented here adds to the relatively sparse literature of high-grade pleomorphic/spindle cell sarcomas arising in less common anatomical sites (e.g., the mandibular region). The imaging features - an expansile lytic lesion with *moth-eaten* margins, linear periosteal reaction, extension into body and ramus of the mandible, and a large soft-tissue component indenting geniohyoid/mylohyoid muscles - strongly suggested aggressive behavior, supporting the need for comprehensive evaluation beyond initial benign impressions. While diagnostic frameworks commonly emphasize histopathology and immunoprofiling, the broader clinical and radiologic context must not be overlooked in directing timely therapeutic decisions. Ultimately, this case underscores that pleomorphic/spindle cell sarcomas may mimic other entities clinically and radiographically, and that multidisciplinary assessment, including imaging, pathology, surgical, and oncologic input, is required for optimal outcome. It further highlights that timely recognition of aggressive radiologic features may expedite definitive management.

## Conclusions

Giant cell-rich lesions of the jaw, such as central giant cell granuloma and giant cell-rich undifferentiated pleomorphic sarcoma, share considerable clinical and histopathologic overlap, particularly on limited biopsy material. However, subtle differences during initial assessment may provide early diagnostic cues. Lesions showing disproportionate radiologic aggressiveness, including cortical destruction, ill-defined margins, and rapid post-extraction progression, should raise concern when such features are discordant with relatively bland or equivocal histopathologic findings. Disproportionate radiologic aggressiveness, clinicopathologic discordance, and tumor heterogeneity should prompt caution in interpreting small biopsy specimens and justify further evaluation.

Management of such lesions should be individualized and guided by definitive histopathology, tumor staging, and multidisciplinary oncologic input. Given the aggressive nature of undifferentiated pleomorphic sarcoma, close postoperative surveillance and long-term follow-up are crucial for early detection of recurrence or metastasis. This report highlights the importance of adequate sampling, integrated diagnostic assessment, and appropriate oncologic management in optimizing patient outcomes in diagnostically challenging giant cell-rich jaw lesions.
